# Defective erythropoiesis in a mouse model of reduced Fbxo7 expression due to decreased p27 expression

**DOI:** 10.1002/path.4571

**Published:** 2015-07-08

**Authors:** Suzanne J Randle, David E Nelson, Shachi P Patel, Heike Laman

**Affiliations:** University of Cambridge, Department of PathologyCambridge, UK

**Keywords:** Fbxo7, ubiquitin ligase, anaemia, cell cycle, mitophagy, differentiation, rs11107

## Abstract

During the final stages of erythropoiesis, lineage-restricted progenitors mature over three to five cell divisions, culminating with withdrawal from the cell cycle and the loss of most organelles, including mitochondria and nuclei. Recent genome-wide association studies in human populations have associated several SNPs near or within *FBXO7* with erythrocyte phenotypes. Fbxo7 encodes a multi-functional F-box protein known to bind p27 and participate in selective mitophagy. One SNP causes an amino acid substitution (Met115Ile) and is associated with smaller erythrocytes. We find that the less common IIe115 allele of Fbxo7 binds less efficiently to p27, and cells expressing this allele proliferate faster than cells expressing Met115. We show that an erythroleukaemic cell line with reduced Fbxo7 expression fails to stabilize p27 levels, exit the cell cycle, and produce haemoglobin. In addition, mice deficient in Fbxo7 expression are anaemic due to a reduction in erythrocyte numbers, and this is associated with lower p27 levels, increased numbers of late-stage erythroblasts with greater than 2*N* DNA content, and delayed mitophagy during terminal differentiation. Collectively, these data support an important physiological, cell cycle regulatory role for Fbxo7 during erythropoiesis. © 2015 Authors. *Journal of Pathology* published by John Wiley & Sons Ltd on behalf of Pathological Society of Great Britain and Ireland.

## Introduction

The daily production of erythrocytes requires massive progenitor cell expansion to generate vast numbers of erythroblasts (EBs), followed by cell cycle withdrawal at the final stages of differentiation, when haemoglobinization and cellular remodelling occur. Careful regulation of the G1 phase is crucial for erythropoiesis, and both pro-proliferative and anti-proliferative activities are essential. This is evidenced in mice with aberrant expression of CDK6, cyclin D3, and cyclin E, which show ineffective erythropoiesis and/or anaemia [1–3]. In addition, removal of organelles, including the mitochondrial network, is part of this differentiation process [4,5]. In erythrocytes, the Nix/BNip3L pathway regulates mitophagy by bridging mitochondria to LC3/GABARAP, recruiting mitochondria into autophagosomes [6]. Knockout mouse models lacking Nix or other autophagy regulators, such as Atg7 or Ulk1, develop anaemia, due to aberrant retention of mitochondria in erythrocytes, increased ROS, and decreased red blood cell (RBC) survival (reviewed in Mortensen *et al* [7]).

F-box proteins (FBPs) confer selectivity to SCF (Skp1–Cullin–FBP)-type E3 ubiquitin ligases, enabling the ubiquitination of recruited substrates. This 69-member family is engaged in a range of activities, many of which are critically important for normal cellular functions [8]. We previously reported alterations in EB numbers in the bone marrow (BM) of mice with a disrupted *Fbxo7* (F-box protein only 7) gene (*Fbxo7^LacZ/LacZ^*) [9]. They showed increased numbers of early-stage EBs and decreased late-stage EBs [9]. More recently, multiple genome-wide association studies (GWASs) have correlated single nucleotide polymorphisms (SNPs) in human *FBXO7* with alterations to RBC parameters, suggesting that FBXO7 affects erythropoiesis [10–12].

FBXO7/PARK15 is implicated in many human diseases, including cancers and early-onset Parkinson's disease [13]. Its involvement in such a variety of diseases indicates that its activities are fundamental in many specialized cells. However, the mechanisms causing dysfunction within individual cell types vary and are not fully understood. FBXO7 is a versatile protein that in addition to being part of an E3 ligase (ubiquitinating proteins such as c-IAP, HURP, and TRAF2) also has non-canonical functions, including acting as a cell cycle regulator by interacting with Cdk6 and p27, and putatively as a regulator of proteasome activity via interaction with PI31. Fbxo7 also regulates stress-induced mitophagy via its direct interaction with Parkin and PINK1 (reviewed in Nelson *et al* [13]). Given the numerous GWASs and reported roles for FBXO7 as a regulator of both G1 phase and mitophagy, we hypothesized that it would regulate erythropoiesis and set out to identify the molecular pathways responsible. Here we report studies of mice with a disrupted *Fbxo7* locus that support an important, physiological, cell cycle regulatory role for Fbxo7 during erythropoiesis.

## Materials and methods

### GST binding assays

*In vitro* binding assays were performed as described previously [14].

### Cell culture

MEL cells were maintained in DMEM, 10% FBS, 2 mM glutamine, 100 U/mL penicillin-streptomycin (Life Technologies, Paisley, Renf, UK). MEL cells were transfected with miR30-based short-hairpin vectors targeting murine *Fbxo7* or empty vector as described [15], or infected using MSCV-based vectors to express human Fbxo7 as described [9]. To induce differentiation, MEL cells were passaged daily in 1.5% DMSO (Sigma, Gillingham, Dorset, UK) at a density of 1 × 10^6^ cells/ml. Haemoglobin quantification using benzidene hydrochloride colorimetric assay was performed as previously described [16]. Proliferation was determined by calculating the log_2_ cell increase, plotted as cumulative population doublings (PD) over time, and inferred using line of best fit.

For blood cultures, 1 µl of EDTA-treated whole blood was cultured with or without 30 µm CCCP (Sigma) or 160 µm bafilomycin A1 in reticulocyte media [17]. Colony-forming assays were performed according to the manufacturer's instructions (StemCell Technologies, Grenoble, France).

### Mice

Animals were housed in accordance with Home Office regulations. Tissue was harvested at 6 weeks, unless otherwise stated. Complete blood counts were performed using a Scil Vet automatic blood counter, and blood smears stained with HemaColor (Merck Millipore, Watford, Herts, UK).

### Flow cytometry

Suppliers of antibodies and dyes were as follows: eBioscience (Hatfield, Herts, UK): CD71-biotin, Ter119-PE, CD48-APC, CD150-PECy7, CD34-APC, FcγRII/III-PECy7, IL7Rα-PECy7, Flk3-PE, CD44-biotin, streptavidin-APC; Life Technologies (Paisley, Renf, UK): biotinylated lineage cocktail (MLM15), c-kit-APC-Cy7, Mac1-biotin, Gr1-PE; BioLegend (London, UK): Sca-1-PB. Mitochondria were stained with 500 nm Mitotracker DeepRed (Invitrogen) for 30 min at 37 °C in complete media. For cell cycle analysis, Click-IT EdU was used (Invitrogen) or for primary cells, 3 × 10^5^ cells were sorted, fixed in 70% ethanol, and stained in 1× PBS, 50 µg/ml PI, 50 µg/ml RNase A.

### Cell fractionation and immunoblotting

For fractionations, equal cell numbers were lysed in 100 µl of RSB buffer (10 mm Tris, pH 7.4; 100 mm NaCl; 25 mm MgCl_2_, 40 µg/ml digitonin; protease and phosphatase inhibitors) for 10 min with rotation. Lysates were passed through a 25 G needle and centrifuged at 13 000 rpm for 15 min. Supernatants were kept as cytosolic fraction, and the pellet was lysed in 50 µl of RIPA buffer with protease and phosphatase inhibitors. Antibodies for immunoblotting were as follows: Santa Cruz Biotechnology (Heidelberg, Germany): CDK6 (sc-177), CDK4 (sc-260), CDK2 (sc-163), p27 (sc-528); Cell Signaling (Danvers, MA, USA): PINK1 (6946), LC3B (2775), GAPDH (2118), Nix (9089), p62 (5114); Sigma: actin, Ponceau S; Abcam (Cambridge, Cambs, UK): Parkin (ab15954), Apotrack; and Fbxo7 as previously described [18].

### Immunoprecipitation (IP) and kinase assays

For kinase assays, 1 × 10^7^ cells were lysed in 500 µl of ELB buffer (50 mm HEPES, pH 7.5; 160 mm NaCl; 5 mm EDTA; 0.1% NP-40, 1× protease inhibitors; 1 mm PMSF), and for co-IPs, 5–10 × 10^6^ cells were lysed in RIPA buffer. For IPs, 1 µg of anti-CDK2 or IgG, with 20 µl of Protein A/G agarose (Santa Cruz Biotechnology), or 20 µl of EZview anti-HA beads (Sigma) was used as previously described [9]. Beads were washed with kinase buffer (25 mm HEPES, pH 7.5; 5 mm MgCl_2_; 2.5 mm MnCl_2_; 0.5 mm DTT) prior to adding 0.75 µg of recombinant 6 × His-Rb (amino acids 792–928; ProSpec, Ness-Ziona, Israel), 37.5 µm ATP, and 1 μCi of [γ-^33^P]ATP for 30 min at 30 °C. Samples were resolved by SDS-PAGE; gels were fixed in 10% acetic acid/methanol, dried, and quantified using a Cyclone Phosphor Imager (PerkinElmer, Waltham, MA, USA).

## Results

### Mice with reduced Fbxo7 are anaemic

**Figure 1:**
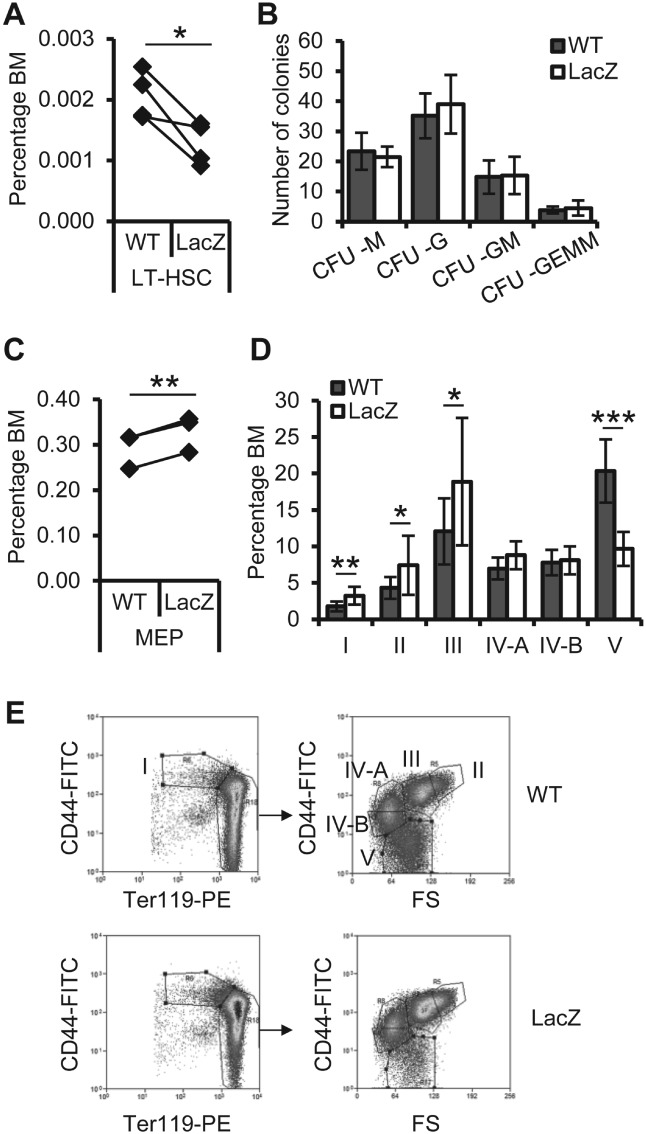
Analysis of stem and multipotent progenitor, and erythroid-specific precursor populations in *Fbxo7^LacZ/LacZ^* mice. (A) Percentage of LT-HSCs (Lineage^−^, Sca-1^+^, c-kit^+^, CD150^+^, CD48^−^) in the BM of wild-type (WT) and *Fbxo7^LacZ/LacZ^* (LacZ) mice. (B) Number of colony-forming units (CFU) per 2 × 10^4^ BM cells after 10 days in culture. CFU-M: macrophage; −G: granulocyte; −GM: mixed granulocyte/macrophage; −GEMM: mixed granulocyte/erythroid/macrophage/megakaryocyte. (C) Percentage of megakaryocyte-erythroid progenitors (MEP; Lineage^−^, Sca-1^−^, c-kit^+^, CD34^−^, FcγRII/III^−^) in the BM. (D) Percentage of EBs in the BM of WT and LacZ animals (*n* = 9), defined using CD44, Ter119, and cell size to differentiate enucleated cells from ortho-EBs, as described by Chen *et al* [20]. I = pro-EBs; II = basophilic EBs; III = polychromatophilic EBs; IV-A = orthochromatophilic EBs; IV-B = reticulocytes; V = mature RBCs. (E) Representative flow cytometry plots of the gating strategy used in D. Ter119^hi^ cells were used for CD44 v forward scatter (FS) analysis. For all figures, values are mean ± standard deviation. *p < 0.05; **p < 0.01; ***p < 0.001.

To characterize erythropoiesis in *Fbxo7^LacZ/LacZ^* mice, we analysed cell populations giving rise to this lineage. Haematopoietic stem cells (HSCs) and multipotent progenitor (MPP) populations within the BM were counted. The percentages of Lineage^−^, Sca-1^+^, c-Kit^+^ (LSK) cells and MPPs arising from long-term HSCs (LT-HSCs), including short-term HSCs, lymphoid-primed MPPs (LMPPs), and common lymphoid progenitors (CLPs), were unchanged (Supplementary Figure 1A). However, LT-HSCs enriched within the LSK, CD150^+^, CD48^−^ compartment [19] were reduced on average by 38% in *Fbxo7^LacZ/LacZ^* mice compared with sex- and litter-matched controls (Figure 1A). Among lineage-defined progenitors, no differences were seen in the common myeloid progenitors (CMPs) and granulocyte/macrophage progenitors (GMPs) by antibody staining (Supplementary Figure 1B) or by methylcellulose assays for myeloid colony-forming units (CFUs) (Figure 1B). There was a significant 12% increase in the percentage of megakaryocytic/erythroid progenitors (MEPs) in *Fbxo7^LacZ/LacZ^* mice (Figure 1C). As previously reported, the number of erythroid progenitors was significantly increased as shown by methylcellulose assays and by antibody staining for CD71 and Ter119, which also showed that *Fbxo7^LacZ/LacZ^* mice had fewer late-stage EBs (independent experiments in Supplementary Figures 1C and 1D) [9]. The CD71^−^, Ter119^hi^ population also contains enucleated reticulocytes and mature RBCs. An alternative staining protocol dependent on CD44, Ter119 expression and cell size [20] can therefore be used to delineate orthochromatophilic EBs (population IV-A) from reticulocytes (IV-B) and RBCs (V). Using this method, we determined that the major population significantly reduced in *Fbxo7^LacZ/LacZ^* mice was mature RBCs (Figures 1D and 1E). This method also identified elevated numbers of erythroid progenitors (populations I, II, and III). The two methods showed a difference with regard to numbers of maturing EBs, which were not different between genotypes using CD71/Ter119 staining (CD71^mid^, Ter119^hi^) (Supplementary Figure 1D), compared with population III using CD44/Ter119/FSC (Figures 1D and 1E). This may reflect the greater overlap of populations I/II/III when CD71 staining is used to delineate this polychromatophilic (poly)-EB population. Nonetheless, both staining protocols indicated an increased number of earlier stage progenitors, yet a reduction in later stage cells including RBCs in the BM of *Fbxo7^LacZ/LacZ^* mice. Importantly, while characterizing EB populations and other tissues in *Fbxo7^LacZ^* mice, we noted that homozygous *LacZ* did not ablate *Fbxo7* expression (Supplementary Figures 1E and 1 F). Therefore, this mouse was not a true null.

**Figure 2:**
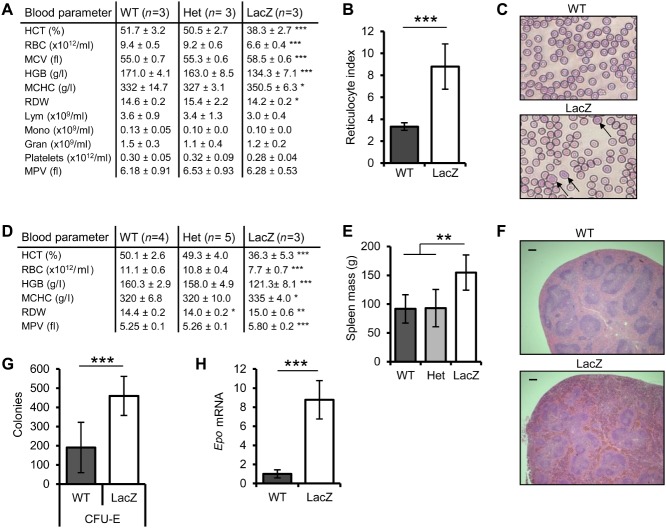
*Fbxo7^LacZ/LacZ^* mice are anaemic. (A) Analysis of blood parameters in 4- to 6-month-old WT, heterozygous (Het), and *Fbxo7^LacZ/LacZ^* (LacZ) mice. HCT = haematocrit; RBC = number of erythrocytes per millilitre of blood; MCV = mean erythrocyte cell volume; HGB = haemoglobin concentration in blood; MCHC = haemoglobin concentration per cell; RDW = red blood cell distribution width; Lym = lymphocyte number; Mono = monocyte number; Gran = granulocyte number; Platelets = platelet number per millilitre of blood; MPV = mean platelet volume. (B) Reticulocyte index (*n =* 9). (C) Blood smears stained with HemaColor, with arrows indicating larger, immature, polychromatophilic cells. (D) Blood counts of 15- to 17-month-old mice. (E) Graph of average spleen mass of WT (*n =* 5), Het (*n =* 8), and LacZ (*n =* 11) mice. (F) H&E-stained spleen sections from WT and LacZ mice showing extramedullary haematopoiesis (bar = 200 µm). (G) Number of erythroid colony-forming units (CFU-E) per 2 × 10^5^ spleen cells after 3 days in culture (*n =* 3, in duplicate). (H) *Epo* mRNA expression in kidneys of WT and LacZ mice relative to cyclophilin A (*n =* 4).

Given the marked reduction in RBCs in the BM, we performed complete blood counts. *Fbxo7^LacZ/LacZ^* mice were anaemic, with a significantly reduced haematocrit (HCT) and a 26% reduction in RBCs (Figure 2A). This decrease correlated with reduced blood haemoglobin (HGB) concentration. In addition, the MCV and concentration of HGB per cell (MCHC) were significantly elevated, with changes in the RBC distribution width (RDW) also evident. Parameters for leukocytes and platelets in *Fbxo7^LacZ/LacZ^* mice were not significantly altered, and heterozygous mice were comparable to WT. *Fbxo7^LacZ/LacZ^* mice also showed reticulocytosis (Figure 2B), evident in blood smears, which showed a high proportion of immature, larger, polychromatophilic cells (Figure 2C, arrows). Anaemia persisted throughout the lifespan of *Fbxo7^LacZ/LacZ^* mice, with additional changes in platelet volume (MPV) becoming apparent by 15 months (Figure 2D).

Anaemia can induce extramedullary haematopoiesis (EMH), and consistent with this, spleens from *Fbxo7^LacZ/LacZ^* mice were 69% larger than those from WT or heterozygous mice (Figure 2E). Extensive EMH was seen histologically in spleen sections, while the white pulp architecture was unperturbed (Figure 2F). When assayed by methylcellulose assays, spleens from *Fbxo7^LacZ/LacZ^* mice had 2.4-fold more erythroid CFUs compared with WT (Figure 2G), as well as elevated numbers of other myeloid CFUs (Supplementary Figure 1G).

Erythropoietin (Epo) levels were assayed in the kidneys by qRT-PCR (Figure 2H). *Fbxo7^LacZ/LacZ^* mice had 8.8 times the amount of *Epo* mRNA compared with littermate controls, eliminating deficient Epo production as a cause of anaemia. However, a defect in Epo signalling cannot be ruled out.

### Fbxo7 stabilizes p27 to enable cell cycle arrest

**Figure 3:**
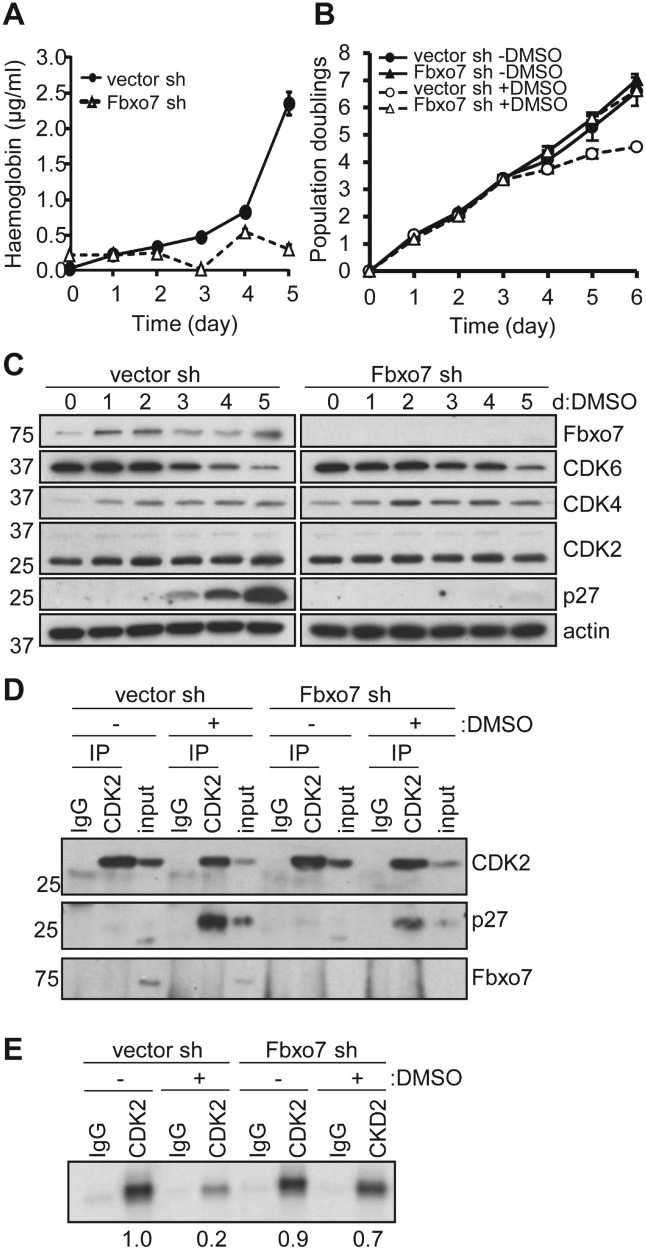
Fbxo7 is required for the differentiation of MEL cells by stabilizing p27 expression. (A) MEL cells expressing an empty vector shRNA (vector-sh) or Fbxo7-specific shRNA vector (Fbxo7-sh) were induced to differentiate over 5 days and the haemoglobin concentration was determined using benzidine colorimetric assay (*n =* 3). (B) Proliferation rate, as measured by population doubling over time, of MEL cells expressing vector-sh or Fbxo7-sh, with (+ DMSO) or without (− DMSO) differentiation-inducing agent (*n =* 3). (C) Immunoblots of whole cell lysates of cells described in B. (D) Immunoblots of anti-CDK2 or IgG immunoprecipitates from MEL cells expressing vector-sh or Fbxo7-sh, with or without 6 days of DMSO treatment. Whole cell lysate prior to immunoprecipitation was used for input. (E) Radiograph of radiolabelled phospho-pRb phosphorylated using immunoprecipitated CDK2 or IgG control, from MEL cells expressing vector-sh or Fbxo7-sh, with or without 6 days of DMSO treatment. Quantification of CDK2 kinase activity, relative to untreated control cells, is below the image.

To investigate the mechanistic basis for the anaemia, we exploited the ability of murine erythroleukaemia (MEL) cells to partially recapitulate erythroid differentiation *in vitro* [21]. Fbxo7 expression was reduced using a previously validated shRNA construct [9]; cells were differentiated by DMSO treatment; and haemoglobin concentration was measured over time (Figure 3A). Control cells accumulated haemoglobin over 5 days, whereas cells expressing *Fbxo7* shRNA (Fbxo7-sh) did not, demonstrating a requirement for Fbxo7 in MEL differentiation.

Proliferation rates during differentiation were also measured (Figure 3B). Fbxo7-sh cells proliferated at the same rate as control cells under normal culture conditions (− DMSO), indicating that Fbxo7 did not affect proliferation. When induced (+ DMSO), control cells slowed proliferation by day 4, while Fbxo7-sh cells continued to divide. The number of cells in the S phase was determined by staining for EdU incorporation and DNA content. In control cells, 35.1% had 2 *N* DNA content (G0/G1 phase) under control conditions, which increased to 71.8% after 6 days of DMSO treatment, indicating G1 arrest. However, Fbxo7-sh cells had similar percentages of cells in the G0/G1 phase, irrespective of treatment (35.2% untreated; 37% DMSO). These data demonstrate that Fbxo7-sh cells failed to arrest cell cycle.

To investigate which cell cycle regulators were affected, cell lysates were immunoblotted (Figure 3C). Neither CDK2 nor CDK4 showed any appreciable differences during differentiation, nor were there any differences between the two cell lines. Down-regulation of CDK6 occurs during MEL differentiation [22], and this was observed in control cells and was largely unaffected by reduced Fbxo7 expression. However, a clear difference in p27 levels was seen: in control cells, p27 expression increased starting at 3 days, but it was absent from Fbxo7-sh cells, indicating that Fbxo7 was necessary for p27 accumulation.

The consequences of a failure to accumulate p27 were investigated by determining its association with CDK2 (Figure 3D). During normal culture, p27 was not detected. When control cells were differentiated, p27 was readily detected bound to CDK2, but this was reduced by 53.5% in Fbxo7-sh cells, when normalized to immunoprecipitated CDK2. This was in line with the 67% reduction in total levels of p27 detected in Fbxo7-sh cells compared with control cells. Fbxo7 was not immunoprecipitated with CDK2, suggesting that it increases the available pool of p27 in cells via independent interactions. We next analysed CDK2 activity using kinase assays (Figure 3E). In DMSO-treated control cells, CDK2 activity decreased by an average of 72% (*p* < 0.001), while Fbxo7-sh cells showed only a 25% reduction in activity after DMSO treatment, which was not significantly different from untreated cells (*p* = 0.08). These data are consistent with a model whereby Fbxo7 stabilizes p27 levels, which inhibits CDK2 and promotes cell cycle withdrawal.

### *Fbxo7^LacZ^^/^^LacZ^* mice have less p27

**Figure 4:**
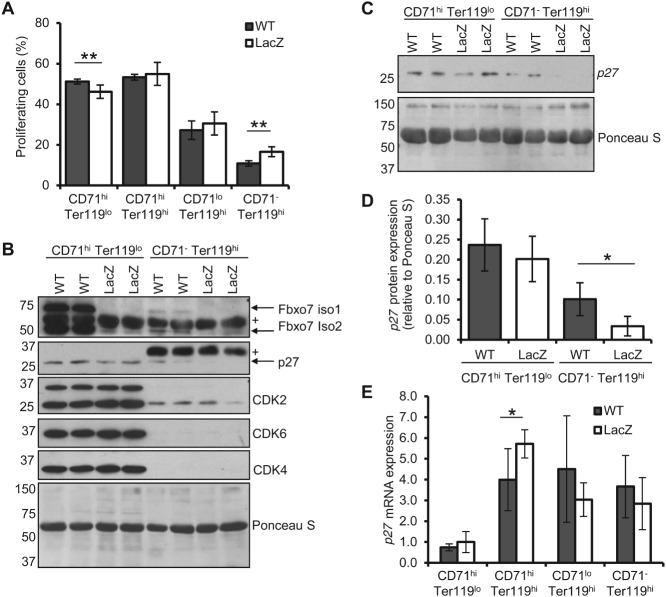
CD71^−^ Ter119^hi^ EBs in *Fbxo7^LacZ/LacZ^* mice have increased numbers of cells with greater than 2 *N* DNA content and less p27. (A) Percentage of cells with greater than 2 *N* DNA content using propidium iodide staining, in EB populations using CD71 and Ter119 staining (WT *n =* 5, LacZ *n =* 4). (B) Immunoblots of whole cell lysates from equal numbers of sorted EBs from WT and LacZ mice. Fbxo7 isoforms 1 (top band) and 2 (bottom band) are expressed. + = non-specific band. Ponceau S was used as total protein loading control. (C) Independent replicate of p27 expression in equal numbers of sorted EBs from WT and LacZ mice. (D) Quantification of p27 expression relative to total protein (Ponceau S) (*n =* 4). (E) Quantitative RT-PCR analysis of *p27* mRNA expression relative to cyclophilin in WT and LacZ mice in sorted EB populations (*n =* 2, in triplicate).

As suggested by the MEL experiments, we next investigated whether a failure to withdraw from the cell cycle was evident in EBs from *Fbxo7^LacZ/LacZ^* mice. To test this, EB populations were sorted using the CD71/Ter119 method, as this gave distinct flow cytometry populations, and the DNA content in nucleated cells was quantified, which excluded reticulocytes and RBCs from analysis. The percentage of EBs with greater than 2 *N* DNA content, suggestive of proliferation, was increased in the late-stage CD71^−^, Ter119^hi^ population in *Fbxo7^LacZ/LacZ^* mice (Figure 4A). Moreover, we found significantly fewer early-stage EBs with greater than 2 *N* DNA content (CD71^hi^, Ter119^lo^), even though they were increased in number in *Fbxo7^LacZ/LacZ^* mice (Supplementary Figure 1D).

We next tested for expression of cell cycle regulators in these sorted populations (Figure 4B). Fbxo7 protein was 71–99% reduced in *Fbxo7*^LacZ/LacZ^ mice (Supplementary Figure 1H). G1 CDKs were all down-regulated during differentiation, but of these, CDK2 was still expressed at the later EB stages. We found that p27 was reduced in late-stage EBs from *Fbxo7^LacZ/LacZ^* mice compared with WT, while no difference was seen in early-stage EBs between genotypes. This was independently repeated (Figure 4C) and quantified to demonstrate there was significantly less p27 in late-stage *Fbxo7^LacZ/LacZ^* EBs compared with WT (Figure 4D). Importantly, *p27* mRNA expression was equivalent between WT and mutant orthochromatophilic EB populations (Figure 4E), indicating that the Fbxo7 effects on p27 were post-transcriptional. Likewise, mRNA expression of the erythroid-specific transcription factor NF-E2 and the p27 transactivator GATA-1 was unchanged between WT and *Fbxo7^LacZ/LacZ^* mice (Supplementary Figures 1I and J1). These data suggest that Fbxo7 stabilizes p27 protein expression, facilitating cell cycle arrest *in vivo*.

### Mitochondrial loss is delayed in *Fbxo7^LacZ^^/^^LacZ^* mice

**Figure 5:**
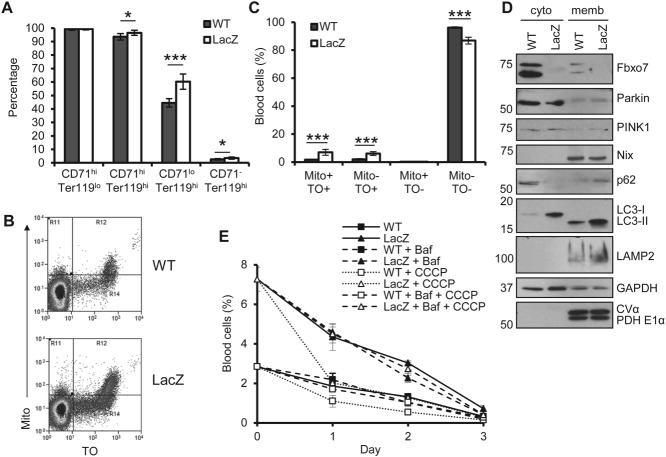
Mitochondrial loss is delayed in *Fbxo7^LacZ/LacZ^* mice. (A) Percentage of Mitotracker DeepRed positive cells in each EB population (*n =* 8). (B) Representative flow cytometry plots of blood cells stained with thiazole orange (TO) and Mitotracker DeepRed (Mito). (C) Percentage of blood cells in each population in B (*n =* 9). Cells are early reticulocytes (TO^+^, Mito^+^), late reticulocytes (TO^+^, Mito^−^), mitochondria-containing RBCs (TO^−^, Mito^+^), and mature RBCs (TO^−^, Mito^−^). (D) Immunoblots of the cytosol (cyto) and membranous (memb) fraction from pooled samples of sorted CD71^hi^, Ter119^hi^ EBs from WT and LacZ mice. Mitochondrial markers, CVα and PDH E1α, are present in the membranous fraction, whereas GADPH is enriched in the cytosolic fraction. (E) Percentage of Mito^+^ cells in whole blood over 3 days in culture, with or without 30 µm CCCP and 160 µm bafilomycin A1 (Baf), from WT and LacZ animals (*n =* 3).

It has been argued that a failure to clear mitochondria can contribute to anaemia. Given the role of Fbxo7 in mediating mitophagy in other settings, we assayed for mitochondrial loss during erythropoiesis by staining BM with Mitotracker DeepRed (Mito), also using the CD71/Ter119 method. Mitochondrial clearance occurred primarily at the later EB stages; however, even from the CD71^hi^, Ter119^hi^ stage onwards, significantly more *Fbxo7^LacZ/LacZ^* EBs had a higher mitochondrial load (Figure 5A). This analysis was also undertaken on peripheral blood, where reticulocytes were analysed using thiazole orange (TO) and Mito (Figure 5B). Compared with WT littermates, *Fbxo7^LacZ/LacZ^* mice had 4.1 times as many reticulocytes with mitochondria (Mito^+^, TO^+^) (Figure 5C).

To investigate this further, CD71^hi^, Ter119^hi^ cells, the first population to show increased mitochondrial load, were purified, separated into membranous and cytosolic fractions, and immunoblotted (Figure 5D). As previously reported, Fbxo7 is predominantly cytosolic and a minority is present in the membranous fraction [14,23]. Consistent with a delay in mitochondrial clearance and altered mitophagic flux, autophagy (p62 and LC3-II) and lysosomal (LAMP2) proteins were elevated in *Fbxo7^LacZ/LacZ^* cells compared with WT. One explanation for this could be that Fbxo7 affects Nix recruitment. However, equal amounts of Nix were detected in the mitochondria-containing fraction from WT and *Fbxo7^LacZ/LacZ^* mice. Likewise, Fbxo7 interactors, Parkin and PINK1, were unchanged in EBs with reduced Fbxo7. These data show that Fbxo7 does not affect the localization of known mitophagy initiators during erythroid differentiation, but mitophagic clearance was either delayed or deficient.

To differentiate between these possibilities, we tested mitochondrial loss in cultured blood (Figure 5E). *Fbxo7^LacZ/LacZ^* mice had significantly more mitochondria-containing cells at day 0, but by day 3, both WT and *Fbxo7^LacZ/LacZ^* cells had lost the majority of their mitochondria. CCCP treatment to stimulate mitophagy caused loss of mitochondria in WT and *Fbxo7^LacZ/LacZ^* cells by day 1 and this was inhibited by bafilomycin A1 treatment, indicating that the mitophagic pathway in these cells was intact. Notably, bafilomycin treatment alone had no effect on mitochondria loss in either WT or LacZ cells, suggesting that additional mitophagy pathways were active. Collectively, these data demonstrate that mitophagy proceeds to completion in *Fbxo7^LacZ/LacZ^* cells and suggest that the increased mitochondrial load seen throughout differentiation is not due to any intrinsic defect. Furthermore, it indicates that reticulocytosis was due to increased/early release of mitochondria-containing reticulocytes from the BM, rather than their accumulation in the periphery, a finding supported by the lack of Mito^+^, TO^−^ cells in the periphery (Figure 5C). These data argue against mitophagic defects underlying the anaemia in *Fbxo7^LacZ/LacZ^* mice.

### Fbxo7 Ile115 binds p27 less efficiently

**Figure 6:**
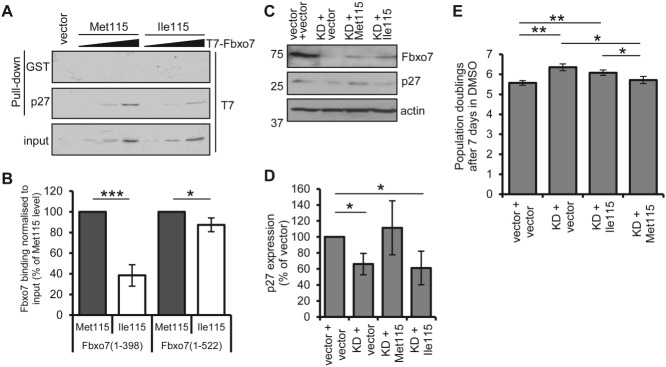
Fbxo7 expressing Ile115 is less able to bind p27. (A) Immunoblots of GST or GST-p27 (1–109) bound T7-Fbxo7 (1–398) expressing Met115 or Ile115. Increasing doses of *in vitro* transcribed and translated Fbxo7 were added to the reaction mixes. (B) Quantification of p27 bound truncated (1–398) or full length (1–522) Fbxo7 expressing Met115 or Ile115, relative to input and expressed as a percentage of Fbxo7 Met115. (C) Immunoblots of MEL cells expressing human Fbxo7 Met115, Fbxo7 Ile115 or empty vector, in control or murine Fbxo7 knockdown (KD) cells. (D) Quantification of p27 protein expression in the cells described in C (*n =* 3). (E) Population doubling of the DMSO-treated cells described in C (*n =* 3).

A GWAS study reported that an SNP (rs11107) causing a Met115Ile change in Fbxo7 is associated with a 0.35-fl decrease in MCV [11]. Given our findings, we tested whether p27 binding was sensitive to this residue in Fbxo7. *In vitro* binding assays were performed with GST-p27 protein, incubated with T7-Fbxo7 with Met115 or Ile115 (Figure 6A). Fbxo7(1–398) Ile115 was 61.5% reduced in p27 binding compared with Fbxo7(1–398) Met115 after normalizing for input (Figure 6B). Similarly, full length Fbxo7(1–522) Ile115 was reduced (12.6%) in binding p27, but the difference was less pronounced, suggesting that the C-terminus stabilized their interaction.

We next tested the effect of the Met115Ile SNP in MEL cells. Endogenous murine Fbxo7, which has Ile115, was reduced using stable expression of shRNA, and human Fbxo7-HA with either Met115 or Ile115, which are resistant to murine shRNA, was then expressed. p27 levels were determined in these cells by immunoblotting (Figure 6C) and then quantified (Figure 6D). We noted that the human Fbxo7 alleles were equivalently expressed in MEL cells, although at a much lower level than endogenous Fbxo7. Importantly, significantly less p27 was found in Fbxo7 KD cells expressing Fbxo7 Ile115 compared with Fbxo7 Met115 (cf. lanes 3 and 4, Figure 6C). Furthermore, the amount of p27 in cells expressing human Fbxo7 Met115 was comparable to the endogenous murine Fbxo7 Ile115 in the control cell line, despite its more abundant expression. This suggests that the Met115 allele of Fbxo7 is much more effective at stabilizing p27 than the human or murine Fbxo7 Ile115 alleles.

These MEL cells were treated with DMSO for 7 days and the number of population doublings (PDs) counted (Figure 6E). Cells with reduced expression of endogenous Fbxo7 (KD + vector) underwent significantly more PDs after DMSO treatment compared with control cells (vector + vector), indicating inefficiency in cell cycle withdrawal. Although human Fbxo7 Ile115 failed to suppress this effect, human Fbxo7 Met115 reduced the number of PDs to a number comparable to control cells. Together, these data demonstrate that Fbxo7 Ile115 binds and stabilizes p27 less well than Met115.

## Discussion

Our study reports on the physiological regulation of erythropoiesis by Fbxo7/PARK15. Importantly, this is a non-canonical function of Fbxo7 and acts via its ability to stabilize p27 levels, and thereby act as a cell cycle regulator. p27 inhibits CDK2, enabling terminally differentiating EBs to exit the cell cycle to form mature RBCs. Erythropoiesis requires the coordination of multiple pathways to enable the production of vast numbers of RBCs over a person's lifetime [24]. It is exquisitely sensitive to even small inefficiencies in the cell cycle, since minor defects amplified at this scale often show dramatic phenotypes. For example, studies on cyclin D3 KO mice showed that 0.7 fewer cell divisions per cell during terminal differentiation led to anaemia [3]. Mice with elevated CDK2 activity due to a hyper-stable cyclin E are also anaemic [2]. The requirement for p27-mediated inhibition of CDK2 activity during erythropoiesis, allowing for cell cycle exit, has been previously reported [25,26]. Here we show that Fbxo7 stabilizes p27 levels to ensure cell cycle arrest and that reduced Fbxo7 expression results in anaemia. Our study eliminates transcriptional effects on p27, GATA1 or NF-E2 as underlying causes for anaemia but as Fbxo7 is a multi-functional E3 ubiquitin ligase, we cannot eliminate the possibility that it may also act via p27-independent mechanisms.

In considering other causes for the anaemia in *Fbxo7^LacZ/LacZ^* mice, we reasoned that Fbxo7 also participates in mitophagy [7], and defects in mitophagy can lead to retention of mitochondria in RBCs, leading to their premature death due to accumulated ROS. Mice with cell cycle defects, such as the hyper-stable cyclin E mouse, and consequential high CDK2 activity, also have defects in mitophagy, which are attributed to a failure to up-regulate Nix [27]. We noted that in *Fbxo7^LacZ/LacZ^* mice, mitochondrial load was higher from the CD71^hi^, Ter119^hi^ (baso-EB) stage onwards and cells had elevated levels of autophagy pathway components, p62, LC3-II, and LAMP2. Although this might suggest defects in mitophagy, reticulocytes from *Fbxo7^LacZ/LacZ^* mice cleared mitochondria under both normal and depolarizing conditions, indicating that mitophagy can proceed efficiently. Moreover, all of the mitophagy regulators tested were expressed and appropriately localized in *Fbxo7^LacZ/LacZ^* EBs. The observed increase in mitochondrial load may stem from a delay in the induction of mitophagy, since this process can be temporally linked to cell cycle withdrawal, which is perturbed in these mice. Alternatively, a failure to down-regulate mitochondrial biogenesis might result in increased load.

CDK6 down-regulation occurs early, initiating the differentiation programme of EBs, which includes gene expression changes [8,21,28,29]. In *in vitro* experiments with MEL cells, we showed that CDK6 was down-regulated in Fbxo7-deficient cells, showing that the Fbxo7 activity promoting differentiation was distinct from CDK6 down-regulation. However, it has been proposed that CDK6 down-regulation alone is not sufficient and that CDK2 inhibition is necessary [28,29], which our experiments support. The cause of increased early-stage EBs in *Fbxo7^LacZ/LacZ^* mice is unknown, but is unlikely to be due to excessive proliferation, since pro-EBs from *Fbxo7^LacZ/LacZ^* mice have fewer cells with greater than 2*N* DNA content than WT cells. It is possible that increased progenitors are a consequence of a block in differentiation at the pro-EB stage. Alternatively, defective cell cycle arrest at later stages *in vivo* may cause a bottleneck effect during differentiation, perhaps with EBs competing for niches within the BM, causing an accumulation of upstream progenitors.

Signs of prolonged anaemia resulting in hypoxia, chronic Epo production, increased erythropoiesis, and EMH, as well as LT-HSC depletion [30], are all present in *Fbxo7^LacZ/LacZ^* mice. Aged mice are also anaemic and show other myeloid changes. We hypothesize that lower LT-HSC numbers may be due to chronic anaemia, although a cell-intrinsic role for Fbxo7 in LT-HSC homeostasis cannot be excluded. It should be noted that mice lacking p27 show no differences in HSC number or erythroid populations [31].

Fbxo7 function in erythropoiesis is clinically relevant, and our study provides a potential mechanism to explain why the Met115Ile SNP is associated with alterations in RBC MCV [11], although our analyses are more difficult due to species differences between human (Met115) and murine (Ile115) Fbxo7. In comparing the human Fbxo7 alleles, we found that the Fbxo7 Met115 was significantly more efficient in binding and stabilizing p27 than Ile115, *in vitro* and in cells. We hypothesize that since it binds p27 less well, humans expressing the minor Fbxo7 Ile115 allele would have decreased p27 levels. Since p27 helps to set the inhibitory threshold for entry into S, a reduction in its levels may lead to a reduced time in G1, which correlates with reduced RBC size. This may manifest as decreased MCV in patients. By contrast, *Fbxo7^LacZ/LacZ^* mice show increased MCV and MCHC parameters in peripheral blood counts. We propose that the ∼90% reduction in Fbxo7 in mutant mice is a more severe phenotype, causing anaemia, than inheritance of rs11107 SNP, which does not. This anaemia is accompanied by EMH and reticulocytosis, and we hypothesize that elevated numbers of larger, immature cells in the peripheral blood contribute to these increased parameters observed in the mouse model.

As can be gleaned from this and other studies, the erythroid lineage is sensitive to cell cycle perturbations. One implication from our study is that, depending on the underlying molecular defect, mild intervention with the cell cycle would enhance RBC production. One area for further study would be to determine whether inhibiting proteins that impact on p27 levels, such as the SCF^Skp2^ ubiquitin ligase that targets its degradation, or, alternatively, Cdk2 inhibition might aid terminal differentiation and improve erythropoiesis. We propose that Fbxo7 should be included in this category, as it impacts on p27 to affect erythropoiesis.

**SUPPORTING INFORMATION ON THE INTERNET**

The following supporting information may be found in the online version of this article:

**Figure S1.** (A) Percentage of LSK (Lineage^−^, Sca-1^+^, c-Kit^+^), ST-HSCs (LSK, CD34^+^, Flt3^−^), and lymphoid-biased progenitor populations (LMPP; LSK, CD34^+^, Flt3^+^, and CLP; Lineage^−^, IL7Rα^+^, Flt3^+^) in BM of wild-type (WT) and *Fbxo7^LacZ/LacZ^* (LacZ) mice. (B) Percentage of myeloid-biased progenitor populations (CMP; Lineage^−^, Sca-1^−^, c-kit^+^, CD34^+^, FcγRII/III^−^, and GMP; Lineage^−^, Sca-1^−^, c-kit^+^, CD34^+^, FcγRII/III^+^) in WT and LacZ mice. (C) Number of (i) mature erythroid burst-forming units (BFU-E) and (ii) erythroid colony-forming units (CFU-E) per 2 × 10^5^ BM cells after 3 days in culture (*n* = 3, in duplicate). (D) Percentage of EBs in WT and LacZ mice based on CD71 and Ter119 staining (*n* = 6). (E) Quantitative RT-PCR analysis of *Fbxo7* mRNA expression (all isoforms) relative to cyclophilin in WT and LacZ mice in sorted EB populations. The number above each bar indicates percentage relative to WT expression in that cell type (*n* = 4). (F) Quantitative RT-PCR analysis of *Fbxo7* mRNA expression (all isoforms) relative to cyclophilin in whole organs from WT and LacZ mice. (G) Number of CFUs per 2 × 10^4^ spleen cells after 10 days in culture. CFU-M: macrophage; −G: granulocyte; −GM: mixed granulocyte/macrophage. (H) Quantification of Fbxo7 protein expression in Figure 4B normalized to Ponceau S and expressed relative to WT isoform 1 expression in CD71^hi^, Ter119^lo^ EBs. (I, J) Quantitative RT-PCR analysis of *GATA1* and *NF-E2* mRNA expression respectively, relative to cyclophilin, in sorted EB populations from WT and LacZ mice.
